# Supplementary material for: Plasma Cell-Free DNA as a Novel Biomarker for the Diagnosis and Monitoring of Atherosclerosis
Source: Cells. 2022 Oct 16;11(20):3248. doi: 10.3390/cells11203248 (PMC9600586; doi:10.3390/cells11203248)
Supplement: Supplementary file 1 [file cells-11-03248-s001.zip › cells-1939595-supplementary.pdf]

Supplemental Figure

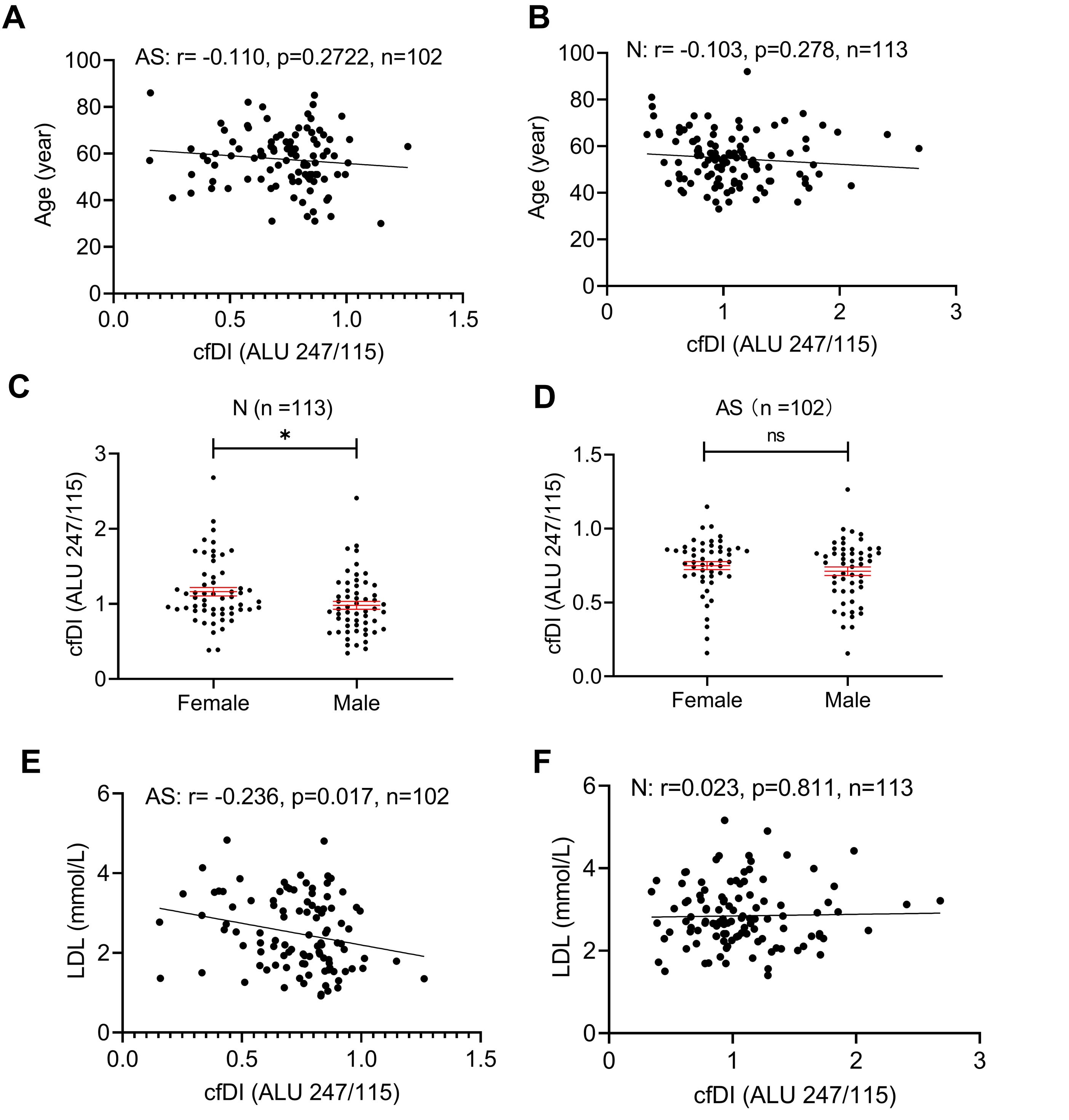

Figure S1. Analysis of association between clinical factors and cfDI. (A-B), correlations of age and cfDI in AS patients (A) and normal controls (B). (C-D), The level of cfDI between different genders in normal group (C) and patient group (D). (E-F), correlations of LDL and cfDI in AS patients (E) and normal controls (F).

Table S1 The basic characteristics of AS patients and normal controls in this study

	N (n=113)	AS (n=102)	P value
Female (%)	56 (49.6%)	50 (49.0%)	
Age (year)	54.8 ± 1.02	57.58 ± 1.20	0.1698
CRP (mg/dl）	1.57 ± 0.37	6.38 ± 1.80	0.02
WBC (*10^9/L)	5.88 ± 0.13	6.55 ± 0.18	0.0029
MONO (*10^9/L)	0.29 ± 0.01	0.45 ± 0.01	<0.0001
NEUT (*10^9/L)	3.5 ± 0.09	3.97 ± 0.16	0.0109
LYMPH (*10^9/L)	1.82 ± 0.05	1.98 ± 0.08	0.0699
TG (mmol/L)	1.57 ± 0.11	2.08 ± 0.17	0.0147
TC (mmol/L)	4.89 ± 0.08	4.34 ± 0.13	0.0002
HDL (mmol/L)	1.26 ± 0.03	1.42 ± 0.10	0.1594
LDL (mmol/L)	2.85 ± 0.07	2.71 ± 0.10	0.2643
Apo A1 (g/L)	1.53 ± 0.04	1.69 ± 0.10	0.3138
Apo B (g/L)	0.91 ± 0.03	1.20 ± 0.12	0.1231
Lpα (ng/L)	137.9 ± 25.45	246.4 ± 22.6	0.0075
HCY(μmol/L)	13.55 ± 0.64	12.67 ± 1.99	0.6022
VB12(pg/mL)	322.6 ± 21.32	501.5 ± 88.39	0.1005
Folate(nmol/L)	11.2 ± 0.91	8.67 ± 0.89	0.0598

CRP, C reactive protein; WBC, white blood cell; MONO, monocyte; NEUT, neutrophil; LYMPH, lymphocyte; TG, triglycerides; TC, total cholesterol; HDL, high-density lipoprotein; LDL, low-density lipoprotein; Apo A1, apolipoprotein A; Apo B, apolipoprotein B; Lpα, Lipoprotein a; Hcy, homocysteine; VB12, vitamin B12.
